# Behavioral restriction, lorazepam, and escitalopram uniquely influence the expression of naturalistic stereotypy in deer mice: perspectives on anxiety- and compulsive-like behavior

**DOI:** 10.3389/fnbeh.2022.1071157

**Published:** 2022-12-19

**Authors:** Johann T. Burke, Daniel C. Mograbi, De Wet Wolmarans

**Affiliations:** ^1^Center of Excellence for Pharmaceutical Sciences, Department of Pharmacology, North-West University, Potchefstroom, South Africa; ^2^Department of Psychology, Pontifical Catholic University of Rio de Janeiro (PUC-Rio), Rio de Janeiro, Brazil; ^3^Institute of Psychiatry, Psychology & Neuroscience, King’s College London, London, United Kingdom

**Keywords:** deer mouse, obsessive-compulsive disorder, stereotypy, restriction, lorazepam, escitalopram, exposure-response prevention

## Abstract

**Introduction:** Stereotypical expression in laboratory-housed rodents can be explained by different motivational, coping, and motor dysfunction theories. Here, we aimed to explore the neurocognitive underpinnings of high stereotypical (HS) expression in deer mice *(Peromyscus maniculatus bairdii)*, previously proposed as a model system of compulsive-like behavioral persistence. Specifically, we aimed to establish whether HS behavior is related to an underlying escape-related trigger.

**Methods:** One-hundred and sixteen deer mice were classified as either non-stereotypical (NS) or HS. Mice of each cohort were further subdivided and exposed to either sub-acute (3-day) or chronic (25-day) behavioral restriction (R), and high-dose escitalopram (ESC), lorazepam (LOR), alone and in combination with R (ESC+R and LOR+R, respectively). Mice were reassessed for stereotypical behavior at both time points.

**Results:** Our results indicate that HS behavior is likely not temporally and functionally related to an anxiogenic trigger, i.e., R, but rather that HS is associated with parallel changes in anxiogenic feedback processing. We also show that chronic R alone significantly decreased the time spent in expressing HS behavior in animals of the HS, but not NS phenotype.

**Discussion:** This points to the possibility that HS-expressing mice represent a subgroup of *P. maniculatus bairdii* in which unique interactions between neurobiology and processes of gradual behavioral organization, may contribute to the expression of the typical behaviors observed in this cohort. Collectively, our findings highlight the value of the deer mouse model system to investigate the potential neurocognitive mechanisms that may underlie the development of persistent phenotypes that can likely not be explained entirely by current theories.

## 1 Introduction

Stereotypical motor expression in laboratory-housed animals sometimes referred to as cage stereotypies (Garner and Mason, 2002), is mostly known to result from environmental deprivation or a lack of environmental complexity (Hadley et al., 2006). In turn, environmental deprivation is known to cause stress and frustration in animals and might narrow the range of available options for behavioral output, thereby contributing to the expression of certain stereotyped behaviors only (Hughes and Duncan, 1988; Eilam et al., 2006). A common difficulty with the conceptual appraisal of stereotypies is related to the underlying meaning of such expression, and thus, it remains difficult to highlight any one suitable explanation for stereotypies that is wholly supported by empirical evidence. This topic has been reviewed extensively before [see Mason and Rushen (2008) for an example]. Suffice to summarize that cage stereotypies can broadly be regarded as coping responses to specific internal (e.g., increased trait anxiety) or external states (e.g., environmental deprivation; Koolhaas et al., 1999), or as motivationally driven behaviors in response to frustration (Würbel, 2006). They can also be viewed as mere motor patterns (or disturbances) that are devoid of an underlying neurocognitive construct (McBride and Parker, 2015). More important than this distinction might be the way these questions are researched since stereotypies are likely to have different meanings within different species and under different circumstances.

In their seminal work on the topic, Würbel et al. (1998) argue that if stereotypies are coping responses to adverse circumstances, such stereotypies will, in the absence of any other external intervention, e.g., improved housing conditions, show rebound after its prevention (that is, when the expression of such behaviors has been prevented for some time); this would indicate motivational build-up over time, much in the same sense as inflated frustration will lead to the expression of stereotypy (Mason and Latham, 2004). The same would conceptually apply to stereotypies that are related to dysfunctional impulses or motor control. On the other hand, if such stereotypies are merely the result of a restricted behavioral repertoire that associate with the formation of behavioral habits, it stands to reason that the expression of such behavior is acquired over time and that it will be attenuated under conditions of prevention (Würbel, 2006). Further to this, in the case of motivationally and coping-related stereotypies, such behaviors will arguably be self-reinforcing, since a lack of lasting positive feedback, e.g., actually escaping from a confined environment, could contribute to a perpetuating cycle of behavioral output (McBride and Parker, 2015). Such a scenario will for obvious reasons not be applicable to stereotypies that are purely founded within the motor domain, or those that are primarily habitual in their output.

Bridging these concepts is a third possibility. Considering that not all animals in a laboratory-housed setting develop spontaneous stereotypy (Hadley et al., 2006; de Ridder et al., 2022), it is possible that subjects displaying high stereotypical (HS) behaviors, might present with a lower sensitivity threshold for stereotypy-triggering stimuli, e.g., anxiety (Koolhaas et al., 1999). From this perspective, stereotypies might be related to distinct implicit processes in certain animals only. In this sense, prevention of stereotypical engagement may over time result in the attenuation of said behavior. We propose two reasons for this view. First, if expressed in response to implicit processes, stereotypy will likely be subject to self-reinforcement (Woody and Szechtman, 2011), a cycle that will be interrupted by the prevention of stereotypical engagement. Second, if stereotypical expression is related to a lower threshold for certain triggers, the prevention of stereotypical expression could result in behavioral adaptation in the face of otherwise unchanging circumstances, much like exposure response prevention (ERP) facilitates compulsive symptom attenuation (McLean et al., 2015).

Against this background, the present research focuses on spontaneous motor stereotypy in deer mice *(Peromyscus maniculatus bairdii)*, which is studied in our laboratory for its resemblance to clinical repetitive behaviors, e.g., compulsivity (Wolmarans et al., 2013). However, a core difficulty with this approach relates to the aforementioned overview, since it is difficult to establish if these behaviors are related to an underlying neurocognitive construct, i.e., being *driven by* an anxiety-related motivational trigger (thus being more akin to a compulsive-like process), or if it is merely representative of a motor disturbance. Here, as before, our arguments lean towards the former. To explain, a brief overview of previous findings in the model system is necessary.

Regarded at face value, the typical stereotypical behaviors expressed by deer mice, i.e., vertical jumping and pattern running, seem to agree with environmental restriction theories since subjects maintained in enriched cages express lower levels of stereotypy (most notably pattern running), which are characterized by a delayed onset and a lower incidence (Powell et al., 1999; Hadley et al., 2006; Bechard et al., 2016). However, stereotypical expression in deer mice varies in terms of its duration and intensity in the same and between different animals over several consecutive assessments, much like the waxing and waning nature of clinical compulsivity (APA, 2013; Wolmarans et al., 2013). It is thus likely that animals could experience motivational, e.g., anxiety-like or escape-driven, build-up prior to expressing stereotypical bouts, which is then followed by bouts during which a relative degree of control is exerted over such behavior. Also, while psychostimulants are known to elicit stereotypical responses and facilitate the transition between goal-directed and habitual responses (Graybiel, 2008; Burguière et al., 2015), vertical activity and horizontal running are not subject to such interference (Tanimura et al., 2009). Also, differences in the social behavior of HS and non-stereotypical (NS) deer mice have been observed which are sensitive to serotonergic intervention (Wolmarans et al., 2017). Thus, HS and NS animals can distinguish between animals of their own cohort and those of the other cohort when placed in a complex social interaction paradigm, meaning that the social interactivity of *P. maniculatus bairdii* is sensitive to the mere observation of stereotypical behavior of other conspecifics. This points to a manner of cognitive appraisal in these animals that facilitates recognition and unique interactions with animals showing similar or different behaviors.

In terms of its response to known anti-compulsive (and anxiolytic) drug interventions, HS, but not NS behavior, is attenuated by serotonergic, but not noradrenergic interventions (Korff et al., 2008; Wolmarans et al., 2013). Such a response is similar to the clinical treatment response of compulsivity. Specifically, high-dose selective serotonin reuptake inhibitor (SSRI) intervention adjusts HS bouts to periods of normal rodent activity and reduces the time that animals spend engaging in HS behavior (Wolmarans et al., 2013). This is despite the fact that animals persist in expressing HS activity during some periods of the dark cycle; such a response is also not unlike that observed in obsessive-compulsive disorder (OCD; Overduin and Furnham, 2012). We have thus argued that SSRIs engender a level of control over the urge to engage in HS behavior (Wolmarans et al., 2013).

Last, the typical behaviors seen in deer mice, are likely less founded on processes related to gradual behavioral shifts and more on a distinct and innate psychobiological profile in HS, compared to NS mice. For example, associations between HS behavior and changes in cognition were shown in our laboratory, whereby HS behavior was associated with lower alternation scores in a T-maze (de Brouwer et al., 2021; de Ridder et al., 2022). This association further seems to be founded upon unique neurobiological underpinnings, as reflected by the response of both HS (but not NS) behavior and T-maze alternation to the cognitive enhancer, levetiracetam (de Ridder et al., 2022) and the anti-adenosinergic drug, istradefylline (de Brouwer et al., 2021). In terms of serotonergic involvement, HS animals show significantly reduced striatal serotonin transporter (SERT) density compared to NS controls (Wolmarans et al., 2013), which is supportive of a hyposerotonergic state in this cohort.

In this work, we aimed to further explore the neurocognitive underpinnings of HS behavior by investigating the role of sub-acute (3 h per day for 3 days) and chronic (12 h per day for 25 days) behavioral restriction on the expression of deer mouse stereotypy. Specifically, we hypothesized that spontaneous HS behavior in deer mice would be related to an inflated degree of escape-related anxiety (or a lower threshold for anxiety-sensitivity) that serves as the self-reinforcing motivational drive for the expression of HS behavior. We further argued that the inability to express said behaviors over the long-term, will abrogate the expression thereof, pointing to HS behavior being sensitive to neurocognitive plasticity on the levels of behavioral control and anxiety processing.

## 2 Materials and Methods

### 2.1 Subjects

Since only 35%–45% of laboratory-housed deer mice (Wolmarans et al., 2013) show H-behavior, an initial pool of 180 deer mice (as far as possible equally distributed between sexes; 10 and 12 weeks old at the onset of experimentation) were bred and sourced from the North-West University (NWU) vivarium (South African Veterinary Council Registration Number: FR15/13458) for initial stereotypical assessment. Randomization occurred according to a numerical system that accounted for litter, rearing cage allocation, and sex. From the first day of experimentation (1 day prior to the onset of the baseline stereotypy assessment; see “2.3 Stereotypical assessment” Section), animals were allocated to new home cages in groups of four same-sex animals and ear-tagged for identification purposes. After the completion of the baseline stereotypy assessment, animals were reallocated to new home cages in pairs of same-sex animals of the same stereotypical cohort and remained so for the duration of the investigation. Cages [35 cm (l)×20 cm (w)×13 cm (h); Techniplast^®^ S.P.A., Varese, Italy) were individually ventilated and maintained at 23°C, with a relative humidity of 55%, and kept on a normal 12-h light/dark cycle (lights on and off at 06:00 and 18:00, respectively; Wolmarans et al., 2013). Food and water (or drug solutions) were available *ad lib* throughout the investigation. Cages were cleaned, and fresh corncob bedding added weekly. Nesting material was provided in the form of a article towel. No other form of environmental enrichment was provided. All experiments were approved by the AnimCare Research Ethics Committee of the North-West University (approval number: NWU-00424-21-A5) and complied with the South African National Standard for the Care and Use of Animals for Scientific Purposes (SANS 10386:2021).

### 2.2 Study layout

**Figure 1:**
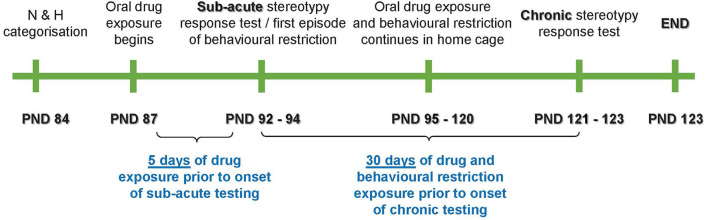
Schematic representation of the study timeline. PND, postnatal day; N, non-stereotypical; H, high stereotypical.

The present work was divided into three distinct stereotypy assessment phases, i.e., at baseline [postnatal day (PND) 84–86], at sub-acute exposure (PND 92–94), and after chronic exposure (PND 121–123; Figure 1).

The main purpose of the sub-acute exposure phase was to assess whether sub-acute behavioral restriction would impact the immediate expression of stereotypical activity against the background of a proposed anxiogenic effect of said restriction. Thus, an anxiolytic control, i.e., the benzodiazepine, lorazepam (LOR), alone and in combination with behavioral restriction (R), was only employed up until the sub-acute phase. However, to determine the long-term response of stereotypy to an anti-compulsive-like control, i.e., the SSRI, escitalopram (ESC), or a combination of ESC and R, animals in these groups were followed through study-endpoint. Therefore, exposures and stereotypical assessments were applied as per Table 1.

**Table 1:** Timeframes for the application of drug exposures, behavioral restriction, and stereotypical assessment.

**Exposure/Restriction**	**Timeframe of application (PND)**	**Sub-acute stereotypical assessment phase**	**Chronic stereotypical assessment phase**
**CTRL**	87–123	92–94	121–123
**LOR**	87–94	92–94	-
**LOR+R**	87–94 (R applied from 92 to 94)	92–94	-
**R**	92–120	92–94	121–123
**ESC**	87–123	92–94	121–123
**ESC+R**	87–123 (R applied from 92 to 120)	92–94	121–123

PND, postnatal day; CTRL, control intervention; LOR, lorazepam; LOR+R, lorazepam and restriction; R, restriction; ESC, escitalopram; ESC+R, escitalopram and restriction.

Control, drug and/or behavioral intervention commenced on PND 87 and continued for 35 days without interruption until the study terminated on PND 123. After the baseline stereotypy assessment, animals of both the NS and HS cohorts were divided into the respective exposure groups as follows: **NS animals**: control (CTRL; *n* = 10; 6 female), ESC (*n* = 10; 6 female), LOR (*n* = 10; 6 female), R (*n* = 8; 4 female), ESC and R combination (ESC+R; *n* = 8; 4 female), and LOR and R combination (LOR+R; *n* = 10; 6 female); **HS animals**: CTRL (*n* = 10; 6 female), ESC (*n* = 10; 6 female), LOR (*n* = 10; 6 female), R (*n* = 10; 2 female), ESC+R (*n* = 10; 2 female), and LOR+R (*n* = 10; 2 female). Thus, the total number of animals included for subsequent investigation was 116. Although all efforts were made to allocate an equal number of male and female animals to each group, breeding and stereotypical selection took place over several weeks to yield the total number of animals needed for the investigation. This resulted in a batch effect that biased some of the groups towards either female or male predominance, a phenomenon that is sometimes reported in this species (Hadley et al., 2006).

### 2.3 Stereotypical assessment

#### 2.3.1 General procedure

Each of the initially included 180 animals underwent three separate 12-h, stereotypy assessment sessions during the dark cycle (PND 84–86) to separate animals into either the NS or HS cohorts. Such an approach, instead of a single assessment, is taken to establish a robust indication of stereotypical activity, given the waxing and waning nature of stereotypy over the course of an assessment session (Wolmarans et al., 2013). On each given assessment day, animals were moved from their housing environment to the behavioral testing room, which was located on the same floor of the vivarium and maintained under conditions identical to that of the housing room. Each mouse was introduced to its own behavioral assessment cage [21 cm (l)×21 cm (w)×35 cm (h); Accuscan Inc., Columbus, USA], constructed from transparent Plexiglas^®^, by 17:00 to allow for habituation to the testing environment prior to the onset of assessment. Each assessment apparatus was fitted with position-detecting infrared (IR) beams that recorded movement every time a beam was interrupted. Beams traversed the cage at both 2 cm (for the recording of pattern running) and 10 cm (for the recording of vertical jumping activity) above floor level. Corncob bedding was provided in quantities enough to cover the floor of the assessment cages but not so that it interrupted the scoring of behavioral data by interfering with the IR beams. Food was provided *ad lib* on the cage floor in the form of broken-up rodent chow pellets. Depending on the phase of behavioral assessment, either normal tap water, ESC, or LOR solutions (see 2.4 “Application of behavioral restriction” Section) were provided through a tight-fit hole in the wall of each cage. To prevent animals from leaving the assessment cages, each apparatus was covered with a transparent lid that allowed for uninterrupted airflow. After each 12-h assessment session, animals were returned to their housing cages. Assessment cages were cleaned with F10^®^ veterinary disinfectant (Health and Hygiene Products^®^, Johannesburg, South Africa). The same procedures were followed during all phases of the investigation.

#### 2.3.2 Analysis of the behavioral data output

Given the waxing and waning nature of deer mouse stereotypy throughout the course of a dark cycle, data generated in the complete 12-h assessment period were divided into 24 individual 30-min bouts. To classify animals as either NS or HS after completion of the first three initial baseline stereotypy assessments, two criteria were applied, i.e., the time spent engaging in stereotypical activity and the intensity of such expression (i.e., the number of stereotypical movements within a specific time bin, that is 30-min). To explain, pattern running and vertical jumping are measured by the number of revolutions an animal makes (expressed as cage revolutions from and ending at the same X-Y coordinate after making a 360° revolution; CR) and the number of vertical beam interruptions (VBI) generated (Wolmarans et al., 2013), respectively. Since mice interrupt more than one beam in the vertical axis when they jump, VBI is applied as a broad measure of jumping activity, rather than being reflective of the actual number of jumps an animal executes. Importantly, although unusual, a single animal can express both behavioral phenotypes. The respective 30-min cut-off values for the classification of NS and HS running and jumping activity are provided in Table 2.

**Table 2:** 30-min cut-off values for the classification of NS and HS running and jumping activity.

**Cohort**	**CR/30 min (Horizontal running count)**	**VBI/30 min (Indicator of jumping activity)**
**NS**	<150	<500
**HS**	>200	>2,000

CR, cage revolutions; VBI, vertical beam interruptions; NS, non-stereotypical; HS, high-stereotypical.

**Figure 2:**
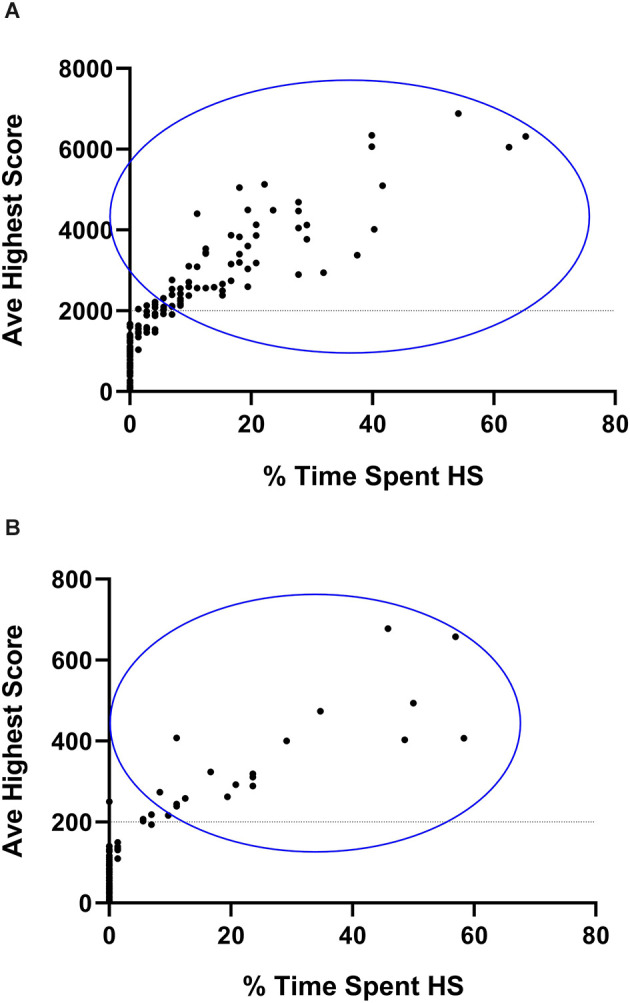
Graphical representation of animals selected for non- (NS) and high stereotypical (HS) behavior. Average highest vertical stereotypy **(A)** and horizontal running **(B)** scores generated by 180 deer mice plotted against the percentage time spent engaging in HS behavior. Y-axis data are representative of the nine highest stereotypical bouts generated over the three baseline assessment sessions. X-axis data are representative of the total number of HS bouts expressed over all 72 baseline bouts. Animals enclosed in the blue circle were included in the HS, some of which may have expressed HS behavior across both phenotypes.

Classification began with calculating the stereotypical intensity. Since CR and VBI are expressed on different scales (hundreds vs. thousands of counts), the average highest stereotypical intensity for each animal, as shown for both the running and jumping phenotypes, was calculated as the average of the nine highest 30-min running and jumping values respectively, as generated by each animal over the course of the complete three-night assessment period. Such an approach is necessary since certain animals show stereotypical expression during a few 30-min bouts over the course of a complete dark cycle only. By regarding the nine highest values (or the three highest values generated per night) as a marker of intensity, a clearer separation between animals that engage in HS, vs. NS behavior is attained, while random variability in the data set is accommodated for. This approach is standard in the present model system (de Brouwer et al., 2021; de Ridder et al., 2022). These averages were then expressed as percentage scores that reflected their deviation from the 30-min cut-off values for the classification of HS behavior. Thus, to calculate the percentage running intensity score for each animal, the formula [($\tilde{x}$ − 200) ÷ 200] × 100 was used, while the formula [($\tilde{x}$ − 2,000) ÷ 2,000] × 100 (where $\tilde{x}$ represents the average of the nine highest 30-min scores generated by each mouse for each phenotype), was applied to represent jumping intensity. The lowest (negative) and highest (positive) value for each animal was subsequently applied to consider NS or HS classification. Second, the average time spent engaging in HS activity was calculated. For this, all HS running and HS jumping bouts respectively, that were generated over the course of all three nights of assessment, were summed and expressed as a percentage out of 72 bouts (i.e., from three 12-h screening sessions, each divided into 24 30-min bouts). Thus, if an animal generated 15 HS running bouts over all three nights, its percentage time spent engaging in HS running behavior would be 20.8% and so forth. The same calculation was applied for the jumping activity and thus, the highest of the two scores for each animal was used. Comparisons between these baseline scores and the subsequent sub-acute and chronic behavioral expression were based on the same calculations. Figure 2 represents animals selected for inclusion in the remainder of the study after their initial selection as explained here.

### 2.4 Application of behavioral restriction

The behavioral restriction was applied over two phases, i.e., during the sub-acute assessment period and over a chronic period of time up until the study endpoint.

With respect to the sub-acute assessment period, animals were for the first time restricted within the testing cages (see “2.3.1 General procedure” Section). Restriction at this time was intended to assess the immediate behavioral response of NS- and HS-expressing mice to acute restriction and how such a response might be modified by anxiolytic intervention. Thus, the restriction was only applied for 3 h during the dark cycle (19:00–22:00) during the three sub-acute assessment sessions on PND 92–94. For this purpose, a Plexiglas^®^ restrictor was inserted into the assessment cage that confined mice to a space of 9 cm (l)×5 cm (w)×8 cm (h). Animals were therefore confined to an area not enabling them to express either running or jumping activity, although normal movement, i.e., grooming and foraging, was otherwise possible. The space in which animals were confined, provided access to food and water (or drug solutions) *ad lib*. Mice were introduced into the assessment cages as per the normal protocol explained above. Periods of restriction were therefore preceded and followed by periods of unrestricted ambulation in the assessment cages. As per “2.2 Study layout” Section, animals were already exposed to drug intervention (ESC and LOR, where applicable) for 5 days prior to the first restriction episode (from PND 87, where applicable) at the onset of the sub-acute assessment phase on PND 92.

For the chronic restriction phase, CTRL-, R-, and ESC+R-exposed animals were restricted for the full dark cycle, every day from PND 95 until PND 120. Since animals resided in home cages in pairs, both animals were confined to the same area in the home cage from 18:00 to 06:00. This area was demarcated by a steel mesh enclosure [18 cm (l)×8 cm (w)×6 cm (h)]. The enclosure was custom-made to allow for uninterrupted access to food and water or drug solutions. From PND 121, animals were again assessed for stereotypical expression for three nights, from and at which time no restriction was applied any longer.

### 2.5 Drug exposure

Escitalopram oxalate [ESC; BLD Pharma^®^, Shanghai, China; 50 mg/kg/day; Wolmarans et al. (2013)] and LOR [Aspen Pharmacare^®^, Qheberha, South Africa; 2 mg/kg/day; Wolmarans et al. (2022)] were prepared for sub-acute (8-day) and chronic (35-day) oral administration *via* the drinking water, respectively. These concentrations were based on the average daily liquid consumption of deer mice [0.25 ml/g/day; Wolmarans et al. (2013) and de Brouwer et al. (2020)]. Drug intake was confirmed by means of daily liquid consumption measurements (average liquid intake of mice receiving normal water, escitalopram, and lorazepam was similar across all days of testing; mixed-effects analysis; inter-day effect: *F*_(29,500)_ = 1.18, *p* = 0.24; between-exposure effect: *F*_(2,23)_ = 1.78, *p* = 0.19; Kruskal-Wallis analysis of the average liquid intake over time per exposure group: *p* = 0.37; average liquid consumption over all days of testing: 4.1 ml per animal; average escitalopram consumption per day: 0.82 ± 0.2 mg; average lorazepam consumption per day: 0.033 ± 0.008 mg). Each cage was supplied with 40 ml of fresh drug solution every day. Oral drug administration *via* drinking water is preferred in this model system, given the anxiogenic potential of intraperitoneal injection and oral gavage. Following the initial classification of animals into the NS and HS groups, drug exposure was initiated. Escitalopram was administered henceforth until the end of the investigation, while LOR was only provided for 8 days, i.e., 5 days prior to and 3 days during the sub-acute stereotypy assessment. Thus, LOR- and LOR+R-exposed animals were euthanized immediately after the sub-acute stereotypy assessment phase, while CTRL-, ESC-, and ESC+R-exposed animals were euthanized at the end of the chronic stereotypy assessment phase only. During the periods of the sub-acute and chronic stereotypy assessment phases, drug solutions were provided in the assessment cages.

### 2.6 Statistical analysis

All analyses and graphical representations were performed and prepared with GraphPad^®^ Prism^®^ version 9.4.1. Behavioral selection of the NS and HS cohorts was conducted as explained in paragraph “Analysis of the behavioral data output”. Thereafter, either normal or repeated measures analysis of variance (2-way ANOVA or 2-way RM-ANOVA) was applied to analyze the stereotypical intensity and the percentage time spent engaging in HS behavior (dependent variables) by animals of the respective exposure groups. These were followed by Bonferroni *post-hoc* analyses for pairwise comparisons of the group means. Time and exposure were set as independent variables. To confirm that animals of the respective exposure groups expressed similar behavior across both dependent variables at baseline, one-way ANOVA with Welch’s corrections was applied. Statistical significance was set at *p* < 0.05 for all analyses.

## 3 Results

### 3.1 Immediate stereotypical intensity post-restriction: sub-acute phase

**Figure 3:**
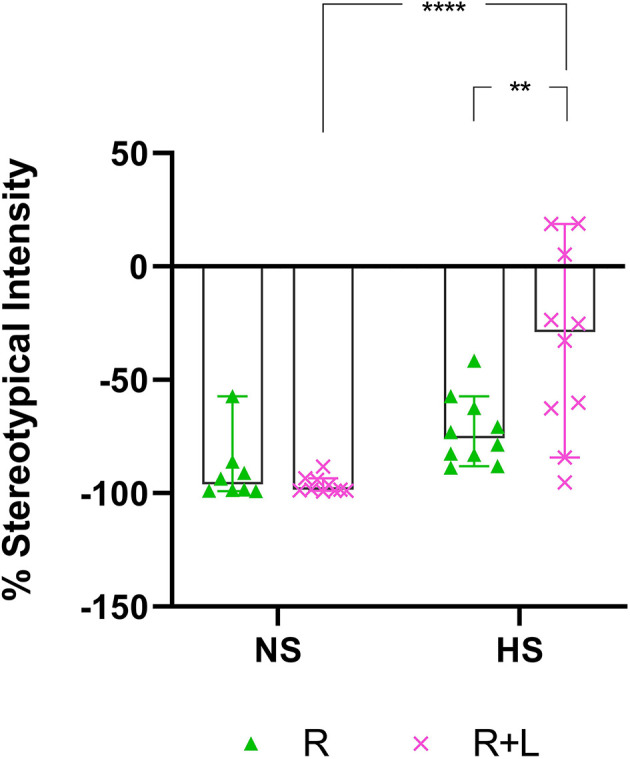
Two-hour post-R stereotypical intensity shown by NS and HS animals in the R and LOR+R groups. 2-way ANOVA followed by Bonferroni’s multiple comparisons tests. Data represent the median ± 95CI. **p < 0.01; ****p < 0.0001; R, behavioral restriction; LOR+R, lorazepam combined with behavioral restriction; NS, non-stereotypical; HS, high stereotypical.

In this comparison, the stereotypical expression of deer mice exposed to either water or LOR was analyzed immediately after the removal of the restrictor in the assessment cage. To this end, the average percentage stereotypical intensity shown by R-, and LOR+R-exposed NS and HS deer mice during the 120-min interval immediately post-restriction over all three assessments during the sub-acute phase, was calculated and compared (Figure 3). The ANOVA results and descriptive statistics for this analysis are provided in Table 3.

**Table 3:** ANOVA results and descriptive statistics: analysis of immediate post-R stereotypical intensity shown by NS and HS mice.

		***NS/HS—R vs. LOR+R***			
		**Mean Difference**	***F* (ANOVA)/Cohen’s *d***	***p***	**95CI of difference**
Figure 3 2 h-Post-Restriction Intensity		***Interaction***			
	Exposure × Phenotype		(1.34) = 8.537	**0.0061**	
		***Main Effects***			
	Exposure		(1.34) = 4.503	**0.0412**	
	Phenotype		(1.34) = 27.53	<**0.0001**	
		***Pairwise Comparisons***			
	*R vs. LOR+R*				
	NS	6.108	0.6 (−1.6; 0.3)	>0.9999	−25.03 to 37.24
	HS	−38.52	**1.2** (0.3;2.2)	**0.0049**	−67.87 to −9.166
	*NS vs. HS*				
	R	−17.76	**1.5** (0.2;2.2)	0.7159	−48.89 to 13.38
	LOR+R	−62.38	**2.1** (1;3.2)	<**0.0001**	−91.74 to −33.03

Bold text indicate significant *p* values.

A significant two-way interaction between exposure and phenotype was shown (*p* = 0.0061). Subsequent *post-hoc* analyses revealed a significant difference between the behavior of NS and HS mice in the LOR+R group (*p* < 0.0001). Further, HS animals exposed to LOR+R expressed significantly higher levels of stereotypy, compared to their R-only-exposed counterparts (*p* = 0.0016). Importantly, most animals of both cohorts (except for some HS animals in the LOR+R group) presented with post-R behavior that failed to meet the criteria for HS activity, as indicated by the distribution of data points in the negative range.

### 3.2 Average highest stereotypical intensity: sub-acute phase

In this analysis, the average highest stereotypical intensity percentages that were generated by animals of the different exposure groups during the remainder of the dark cycle, i.e., all bouts excluding the time during which behavioral restriction was applied, were compared. For this analysis, data from all three nights were pooled.

**Figure 4:**
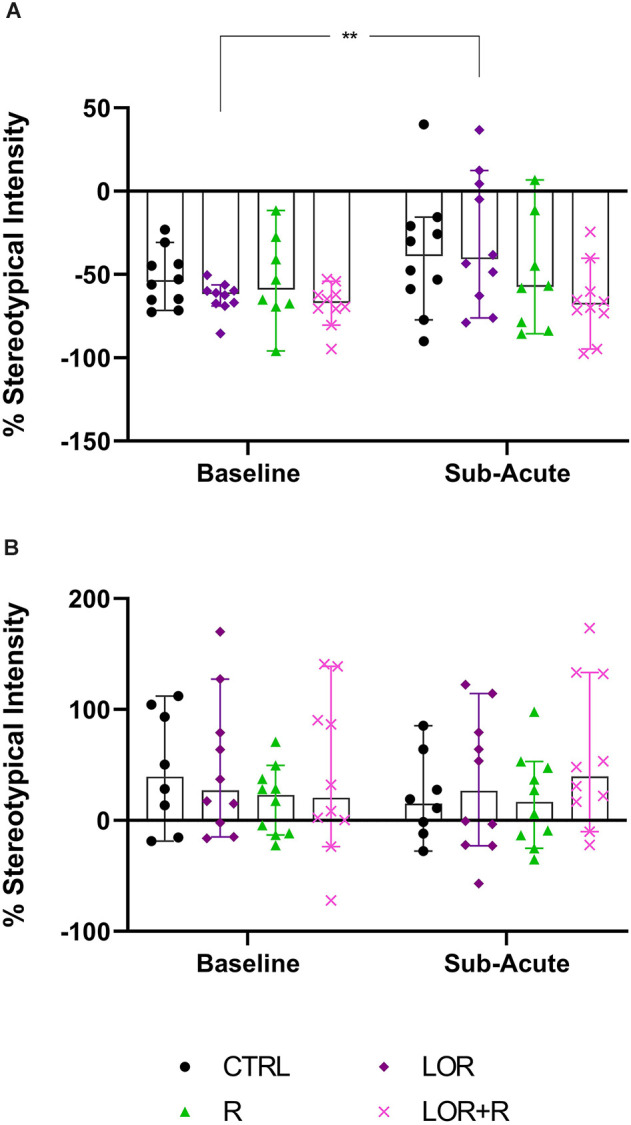
Average whole-night stereotypical intensity shown at the sub-acute phase in **(A)** NS and **(B)** HS mice exposed to CTRL, R, L, and LOR+R. 2-way RM ANOVA followed by Bonferroni’s multiple comparisons tests. Data represent the median ± 95CI. **p < 0.01; CTRL, control (drug- and restriction-naïve animals); R, behavioral restriction; L, lorazepam; LOR+R, lorazepam combined with behavioral restriction.

In the behavior of NS animals (Figure 4A; Table 4), no significant two-way exposure-time interaction was shown. Rather, time impacted the result in a significant manner (*p* = 0.0169). Here, a significant increase in the stereotypical intensity shown by NS animals exposed to LOR was shown (*p* = 0.0083). For HS animals (Figure 4B; Table 4), no interaction effect, nor any main effects of exposure and time, were shown.

**Table 4:** RM-ANOVA results and descriptive statistics: analysis of average whole-night stereotypical intensity and time spent engaging in HS behavior at both the sub-acute and chronic study phases.

		**NS**				**HS**			
		**Mean difference**	***F* (ANOVA)/Cohen’s *d***	***p***	**95CI of difference**	**Mean difference**	***F* (ANOVA)/Cohen’s *d***	***p***	**95CI of difference**
Figure 4 Sub-Acute Phase Ave. stereotypy intensity		***Interaction***				***Interaction***			
	Exposure × Time		(3.34) = 2.107	0.1176			(3.34) = 0.6385	0.5955	
		***Main Effects***				***Main Effects***			
	Exposure		(3.34) = 2.211	0.1047			(3.34) = 0.9297	0.4369	
	Time		(1.34) = 6.315	0.0169			(1.34) = 0.2331	0.6323	
		***Pairwise Comparisons***				***Pairwise Comparisons***			
	*Sub-Acute Exposure*								
	CTRL vs. LOR	−7.970	0.2 (−0.7; 1.1)	>0.9999	−40.42 to 24.48	−11.99	0.2 (−0.7; 1.1)	>0.9999	−83.29 to 59.31
	CTRL vs. R	13.63	0.4 (−1.3; 0.6)	>0.9999	−20.80 to 48.05	2.226	0.1 (−0.9; 0.8)	>0.9999	−69.07 to 73.53
	CTRL vs. LOR+R	28.43	**0.9** (−1.9; 0)	0.1205	−4.022 to 60.88	−36.97	0.7 (−0.2; 1.6)	0.9799	−108.3 to 34.33
	*Baseline vs. Sub-Acute*								
	Ctrl	−14.62	0.5 (−0.4; 1.4)	0.6401	−41.48 to 12.23	25.21	0.5 (−1.4; 0.4)	>0.9999	−40.57 to 91.00
	LOR	−33.95	**1.1** (0.2; 2.1)	**0.0083**	−60.80 to −7.098	14.87	0.2 (−1.1; 0.6)	>0.9999	−43.97 to 73.71
	R	−2.322	0.1 (−0.9; 1.1)	>0.9999	−32.34 to 27.70	−0.5315	0.01 (−0.86; 0.89)	>0.9999	−59.37 to 58.31
	LOR+R	−1.846	0.1 (−0.8; 1.0)	>0.9999	−28.70 to 25.01	−17.34	0.2 (−0.6; 1.1)	>0.9999	−76.19 to 41.50
Figure 5 Sub-Acute Phase Ave. time spent engaging in HS behavior		***Interaction***				***Interaction***			
	Exposure × Time		(3.34) = 1.203	0.3235			(3.34) = 0.2515	0.8597	
	Exposure		(3.34) = 0.9885	0.4098			(3.34) = 2.877	0.0503	
	Time		(1.34) = 7.599	**0.0093**			(1.34) = 0.4563	0.5039	
		***Pairwise Comparisons***				***Pairwise Comparisons***			
	*Sub-Acute Exposure*								
	CTRL vs. LOR	−4.189	0.5 (−0.4; 1.3)	0.5227	−10.75 to 2.369	7.105	0.5 (−1.4; 0.4)	>0.9999	−9.334 to 23.54
	CTRL vs. R	−1.558	0.2 (−0.7; 1.2)	>0.9999	−8.514 to 5.397	7.287	0.5 (−1.4; 0.4)	>0.9999	−9.152 to 23.73
	CTRL vs. LOR+R	1.600	0.4 (−1.3; 0.5)	>0.9999	−4.958 to 8.157	−0.5046	0.03 (−0.8; 0.9)	>0.9999	−16.94 to 15.93
	*Baseline vs. Sub-Acute*								
	Ctrl	−1.775	0.5 (−0.4; 1.4)	>0.9999	−8.053 to 4.503	5.482	0.4 (−1.3; 0.5)	>0.9999	−11.02 to 21.98
	LOR	−7.016	**0.8** (−0.1; 1.7)	**0.0230**	−13.29 to −0.7378	2.982	0.2 (−1.1; 0.7)	>0.9999	−11.77 to 17.74
	R	−3.508	0.7 (−0.3; 1.8)	0.7848	−10.53 to 3.511	−1.398	0.2 (−0.7; 1.1	>0.9999	−16.15 to 13.36
	LOR+R	−1.228	0.6 (−0.3; 1.5)	>0.9999	−7.506 to 5.051	0.7247	0.04 (−0.9; 0.8)	>0.9999	−14.03 to 15.48
Chronic Phase Ave. stereotypy intensity		***Interaction***				***Interaction***			
	Exposure × Time		(6.64) = 1.157	0.3405			(6.64) = 0.9217	0.4854	
		***Main Effects***				***Main Effects***			
	Exposure		(3.32) = 1.873	0.1541			(3.32) = 1.841	0.1596	
	Time		(1.596. 51.08) = 2.104	0.1416			(1.771.56.66) = 2.998	0.0639	
		***Pairwise Comparisons***				***Pairwise Comparisons***			
	*Sub-Acute Exposure*								
	CTRL vs. ESC	9.021	0.3 (−1.2; 0.6)	>0.9999	−37.84 to 55.89	34.64	0.5 (−1.5; 0.3)	>0.9999	−75.11 to 144.4
	CTRL vs. R	9.155	0.2 (−1.2; 0.7)	>0.9999	−51.76 to 70.07	22.99	0.5 (−1.4; 0.4)	>0.9999	−67.43 to 113.4
	CTRL vs. ESC+R	−3.287	0.1 (−0.8; 1.0)	>0.9999	−63.09 to 56.52	−17.33	0.3 (−0.6; 1.1)	>0.9999	−127.1 to 92.39
	*Chronic Exposure*								
	CTRL vs. ESC	39.45	**0.8** (−1.8; 0.1)	>0.9999	−37.97 to 116.9	53.04	**1.0** (−2.0; −0.1)	0.8829	−36.27 to 142.3
	CTRL vs. R	39.02	**0.8** (−1.7; 0.2)	>0.9999	−40.59 to 118.6	58.97	**1.4** (−2.5; −0.5)	0.1691	−12.52 to 130.5
	CTRL vs. ESC+R	15.35	0.3 (−1.2; 0.6)	>0.9999	−66.15 to 96.85	39.06	0.7 (−1.6; 0.2)	>0.9999	−53.43 to 131.5
	*Baseline vs. Sub-Acute*								
	Ctrl	−17.37	0.6 (−0.3; 1.5)	0.1612	−40.34 to 5.602	18.44	0.3 (−1.2:0.6)	>0.9999	−72.97 to 109.8
	ESC	−21.29	**1.0** (0.1; 1.9)	0.0162	−38.44 to −4.137	31.15	0.6 (−1.5; 0.3)	0.6284	−39.35 to 101.7
	R	−2.993	0.1 (−0.9; 1.1)	>0.9999	−47.05 to 41.07	11.49	0.4 (−1.3; 0.5)	0.6561	−14.00 to 36.99
	ESC+R	−9.974	0.3 (−0.7; 1.3)	>0.9999	−42.55 to 2.60	−12.74	0.2 (−0.7; 1.0)	>0.9999	−73.81 to 48.34
	*Baseline vs. Chronic*								
	Ctrl	−34.82	**0.8** (−1; 1.7)	0.3446	−93.33 to 23.70	3.376	0.1 (−0.9; 0.8)	>0.9999	−105.2 to 112.0
	ESC	−8.314	0.4 (−0.5; 1.3)	>0.9999	−32.60 to 15.98	34.49	0.7 (−1.6; 0.2)	0.6078	−42.24 to 111.2
	R	9.423	0.3 (−1.3; 0.7)	0.9819	−18.56 to 37.41	32.40	**1.0** (−2.0; −0.1)	0.0244*	4.279 to 60.53
	ESC+R	−8.788	0.3 (−0.7; 1.3)	>0.9999	−58.76 to 41.19	28.59	−0.4 (−1.3; 0.5)	0.6096	−32.52 to 89.71
Figure 6 Chronic Phase Ave. time spent engaging in HS behavior		*Interaction*				*Interaction*			
	Exposure × Time		(6.64) = 1.525	0.1844			(6.64) = 1.973	0.0826	
		***Main Effects***				***Main Effects***			
	Exposure		(3.32) = 2.432	0.0831			(3.32) = 5.529	**0.0036**	
	Time		(1.354.43.34) = 2.856	0.0867			(1.739.55.66) = 2.608	0.0898	
		***Pairwise Comparisons***				***Pairwise Comparisons***			
	*Sub-Acute Exposure*								
	CTRL vs. ESC	0.8450	0.3 (−1.2; 0.6)	>0.9999	−4.200 to 5.890	13.19	**1.0** (−1.9; −0.1)	>0.9999	−11.83 to 38.22
	CTRL vs. R	−2.150	0.4 (−0.5; 1.4)	>0.9999	−11.66 to 7.358	7.997	0.6 (−1.5; 0.3)	>0.9999	−16.71 to 32.70
	CTRL vs. ESC+R	−1.737	0.4 (−0.5; 1.4)	>0.9999	−8.502 to 5.028	3.375	0.2 (−1.1; 0.7)	>0.9999	−22.18 to 28.93
	*Chronic Exposure*								
	CTRL vs. ESC	6.390	**0.8** (−1.8; 0.1)	>0.9999	−6.849 to 19.63	28.08	**1.4** (−2.4; −0.4)	0.3258	−11.69 to 67.84
	CTRL vs. R	5.904	0.7 (−1.7; 0.2)	>0.9999	−7.261 to 19.07	30.26	**1.5** (−2.6; −0.6)	0.2250	−10.21 to 70.73
	CTRL vs. ESC+R	1.563	0.2 (−1.1; 0.8)	>0.9999	−13.17 to 16.30	24.93	**1.2** (−2.2; −0.3)	0.5744	−14.66 to 64.51
	*Baseline vs. Sub-Acute*								
	Ctrl	−1.401	0.5 (−0.4; 1.2)	0.5462	−4.244 to 1.441	8.333	0.5 (−1.5; 0.3)	0.4959	−10.45 to 27.12
	ESC	−0.8340	0.5 (−0.4; 1.4)	0.3341	−2.220 to 0.5520	10.06	**0.8** (−1.7; 0.1)	0.3501	−8.886 to 29.01
	R	−3.343	0.7 (−0.3; 1.7)	0.5476	−10.41 to 3.723	1.663	0.2 (−1.1; 0.7)	>0.9999	−6.414 to 9.740
	ESC+R	−2.583	**0.8** (−0.2; 1.8)	0.4110	−7.392 to 2.227	2.786	0.2 (−1.0; 0.7)	>0.9999	−9.596 to 15.17
	*Baseline vs. Chronic*								
	Ctrl	−6.112	**0.8** (−0.1; 1.8)	0.3097	−16.00 to 3.777	−6.896	0.3 (−0.6; 1.2)	>0.9999	−37.13 to 23.34
	ESC	−0.0003333	0 (−0.9; 0.9)	>0.9999	−1.359 to 1.358	9.716	0.7 (−1.7; 0.1)	0.3729	−9.133 to 28.57
	R	0.0004584	0 (−1.0; 1.0)	>0.9999	−2.959 to 2.960	8.699	**1.5** (−2.6; −0.5)	**0.0020**	3.829 to 13.57
	ESC+R	−3.994	0.7 (−3; 1.7)	0.6144	−12.93 to 4.941	9.109	0.5 (−1.4; 0.4)	0.2766	−5.929 to 24.15

Bold text indicate significant *p* values.

### 3.3 Time spent engaging in HS activity: sub-acute phase

**Figure 5:**
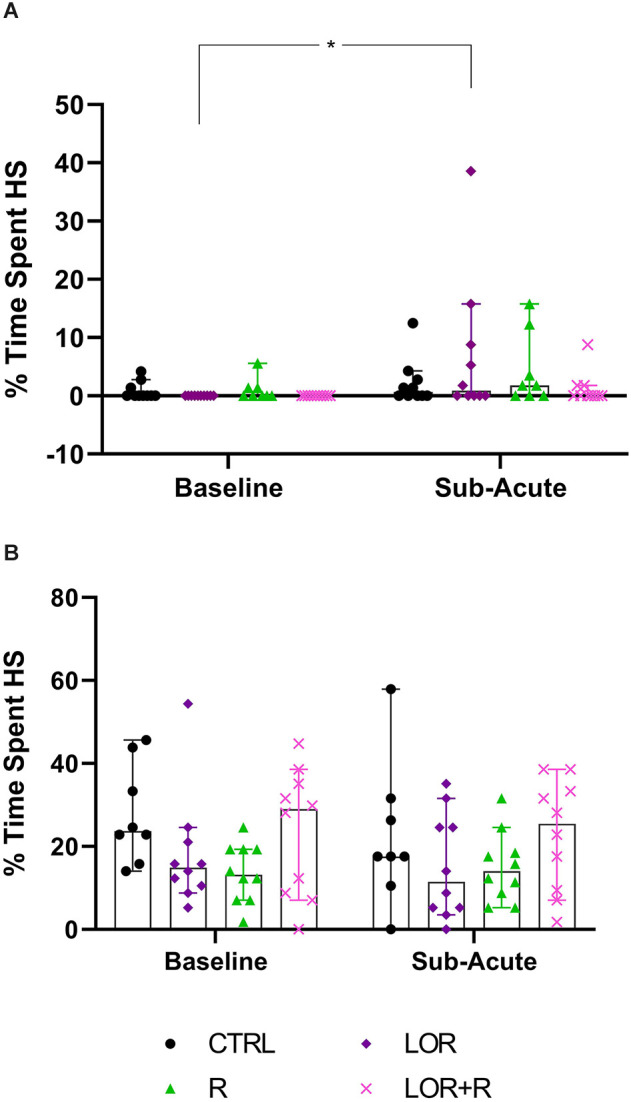
Average time spent engaging in HS behavior at the sub-acute phase by **(A)** NS and **(B)** HS mice exposed to CTRL, R, L, LOR+R. 2-way RM ANOVA followed by Bonferroni’s multiple comparisons tests. Data represent the median ± 95CI. *p < 0.05; CTRL, control (drug- and restriction-naïve animals); R, behavioral restriction; L, lorazepam; LOR+R, lorazepam combined with behavioral restriction.

Here, the total time spent engaging in HS behavior by both NS (Figure 5A; Table 4) and HS animals (Figure 5B; Table 4) of the different exposure groups during the remainder of the dark cycle, i.e., all bouts excluding the time during which behavioral restriction was applied, was compared. For this, data from all three nights were again pooled.

For NS animals, no significant exposure-time interaction was shown. Again, time impacted the result in a significant way (*p* = 0.0096), with a difference observed between the baseline and sub-acute values generated by mice exposed to LOR (*p* = 0.0174). HS animals did not respond to either exposure or time, with no significant two-factor interaction, nor any main effect of exposure or time is shown.

### 3.4 Average highest percentage stereotypical intensity over time: sub-acute and chronic phases

In this analysis, the average highest stereotypical intensity percentages that were generated by NS (Table 4) and HS animals (Table 4) of the different exposure groups during the sub-acute (remainder of the non-restricted times in the dark cycle) and chronic (whole night) assessment phases were compared. For this, data from all three nights during the sub-acute and chronic assessment phases, respectively, were pooled.

No significant interaction between exposure and time, nor significant main effects of exposure or time was shown with respect to the behavior of NS animals. The same was found for the behavior of HS animals, although a decreasing trend over time was observed in the behavior of ESC- and R-exposed animals.

### 3.5 Time spent engaging in HS activity over time: sub-acute and chronic phases

**Figure 6:**
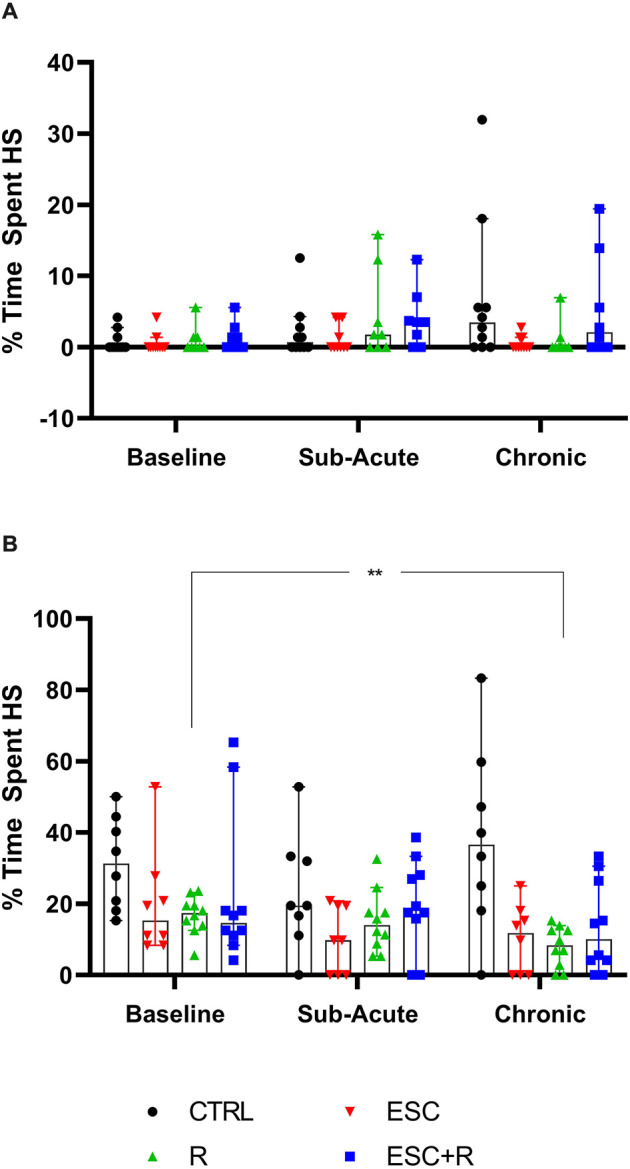
Average time spent engaging in HS behavior at the chronic phase by **(A)** NS and **(B)** HS animals exposed to CTRL, R, ESC, and ESC+R. 2-way RM ANOVA followed by Bonferroni’s multiple comparisons tests. Data represent the median ± 95CI. CTRL, control (drug- and restriction-naïve animals); R, behavioral restriction; ESC, escitalopram; ESC+R, escitalopram combined with behavioral restriction. **p < 0.01.

Here, the total time spent engaging in HS behavior by both NS (Figure 6A; Table 4) and HS animals (Figure 6B; Table 4) of the different exposure groups during the sub-acute (remainder of the non-restricted times in the dark cycle) and chronic (whole-night) assessment phases were compared. For this, data from all three nights during the sub-acute and chronic assessment phases, respectively, were also pooled.

In the absence of a two-factor interaction, neither exposure nor time pies in laboratory housed influenced the manner in which NS-expressing mice engaged in stereotypical activity. However, with respect to the behavior of HS animals, for which a two-way interaction was also not shown, exposure significantly impacted the result recorded (*p* = 0.0036), with a significant reduction in the baseline time spent engaging in HS behavior recorded for R-exposed animals, at study endpoint (*p* = 0.0020).

## 4 Discussion

In this work, we highlight three major findings. First, mice classified as NS and HS that were exposed to behavioral R, alone or in combination with LOR, responded distinctly immediately post-R. Specifically, HS animals in the LOR+R group presented with significantly higher levels of post-R stereotypy compared to NS animals of the same exposure group, and compared to HS animals that were only subjected to R. Second, sub-acute LOR, but not ESC or R, induced expression of HS behavior in NS animals, without further exacerbating the behavior of HS mice. Third, chronic, but not sub-acute R induced a significant and robust decrease in the expression of HS behavior in animals classified as HS.

The present work was conducted to further explore the neurocognitive underpinnings of spontaneous stereotypy in deer mice, previously proposed to be a model of compulsive-like behavior (Wolmarans et al., 2013). Spontaneous cage stereotypies in laboratory-housed rodents are common and different theories are proposed to explain them. Of specific relevance for translational investigations into compulsive-like processes, are theories that ascribe the expression of stereotypy to a behavioral coping need (Mason, 1991) or to an underlying motivational drive (Würbel, 2006). Taking this into consideration and given the resemblance of deer mouse stereotypy to clinical compulsive neurocognitive characteristics (Scheepers et al., 2018), the “functional” purpose and underlying triggers of HS, as opposed to NS behavior, remains to be established in the present model system.

In terms of the first two main findings of this work, sub-acute behavioral R in combination with LOR had the opposite effect on the immediate post-R behavior of NS and HS animals. While R on its own blunted the immediate post-R expression of stereotypy in both phenotypes (as reflected by the negative stereotypy intensity scores generated by animals of both phenotypes), concurrent LOR exposure reversed this suppression in HS, but not NS deer mice (Figure 3). The restriction of “normal” movement is a known anxiogenic stressor that is often investigated and applied in animal models of stress-related behaviors (Beerda et al., 1999; Tilbrook et al., 2000; Morgan and Tromborg, 2007). Here we show that LOR significantly intensified the post-R stereotypical expression of HS mice. It could be argued that a similar neurobiological effect of LOR on R-induced anxiety in NS animals may be masked, due to NS animals not spontaneously presenting with high levels of stereotypy. That said, such a conclusion is unlikely, given the drug-specific increase observed in the average whole-night stereotypical expression of NS animals exposed to LOR in the absence of R (Figure 4A). Rather, we propose an alternative explanation of this result that is founded upon the potential influences of arousal states on the expression of disinhibited behavior (Jones et al., 2013). To explain, considering that both NS and HS animals present with stereotypical behaviors, albeit to different degrees, and that LOR administered on its own elicited stereotypical expression in NS animals during the remainder of the dark-cycle post-R, it is possible that differences in anxiety-processing biology in NS and HS animals could have differentially modulated the effects of LOR in this model system. We have previously shown that LOR elicits a behavioral response in this species (albeit in compulsive-like large-nesting subjects under conditions of open-field stress), that was proposed to be reminiscent of behavioral disinhibition (Wolmarans et al., 2022). Here, we propose that under conditions of acute stress, i.e., R, such a response is seen in animals of the HS phenotype, while a similar response to LOR is only seen in NS animals in the absence of an acute stressor (Figure 4); conversely, under the latter conditions, LOR has no effect on the behavioral output of HS animals. Earlier work investigating behavioral disinhibition in drug- (Constantinou et al., 2010) and alcohol users (Zack et al., 2011) elegantly showed that high states of emotional arousal, as is true for anxiogenic circumstances, can either blunt or increase behavioral disinhibition. If we consider then that NS and HS animals may present with unique anxiety-like behavioral profiles (Wolmarans et al., 2022), it is possible that differences in the anxiogenic valence of R-related feedback in NS and HS mice could have uniquely influenced the manner in which LOR elicited the observed responses. Benzodiazepines are known to cause behavioral disinhibition in a context-sensitive manner (Paton, 2002), a phenomenon that has been shown in both pre-clinical (Ferrari et al., 1997) and clinical (Weisman et al., 1998) settings. For example, the benzodiazepine, chlordiazepoxide, was previously shown to bolster aggressive behavior only in animals prior selected for aggressive responses, while Weisman et al. (1998) showed that healthy individuals showed signs of benzodiazepine-induced behavioral disinhibition under conditions of mild provocation, only. The present findings are in support of this view, since we also show an acute response of deer mouse behavior to R-induced anxiety (Figure 3), while the effects of LOR seem to diverge on the level of behavioral phenotype (Figure 3 vs. Figure 4; response of NS animals immediately post-R vs. the whole-night response). In terms of our hypothesis that a functional and temporal (or motivational) relationship between an underlying sense of escape-related anxiety and the expression of stereotypical behavior exists, our present findings are insightful. Given that the 3-h R period did not elicit an immediate post-R increase in stereotypical expression in animals of either cohort, it can be concluded with a relative degree of certainty that stereotypy in deer mice is not directly and temporally related to mounting anxiety. Considering the coping and motivational theories of stereotypy, it could have been expected that mounting frustration at not being able to express stereotypical behavior, would have resulted in the rebound expression of stereotypical responses in HS animals (Mason and Rushen, 2008). This did not transpire. Rather, our findings are likely providing indirect perspectives on potential differences in the limbic processing of NS and HS mice, which may associate with, but not be directly related to the expression of stereotypy.

With respect to the last main finding of this work, our results are somewhat incongruent with earlier findings regarding the effects of ESC in this model system (Wolmarans et al., 2013). That ESC did not affect the average highest stereotypical intensity of NS and HS deer mice after chronic administration (Table 4) was expected and is in line with our previous work showing that HS animals persist in the expression of HS behavior after chronic serotonergic intervention. However, our earlier result that showed ESC to reduce the time spent engaging in stereotypy, could not be replicated here (Wolmarans et al., 2013). Although the same trend was observed in the present work, the fact that such a response was observed at both the sub-acute and chronic stages of the investigation, undermines a conclusion that ESC exerts its maximum effect on stereotypy after chronic administration only (Wolmarans et al., 2013). An explanation for this unexpected finding might be related to the manner of investigation in the present work. In our earlier work, deer mice were housed singly with each animal acting as its own treatment-naïve control prior to the initiation of ESC exposure (Wolmarans et al., 2013). Further, to track the response of stereotypy over time, each animal was screened for stereotypy weekly for 5 weeks. Thus, we cannot exclude the possibility that habituation to the testing environment at the time, could have interacted with the effect of ESC on the expression of HS behavior. To this, we afforded detail in the relevant article. Still, the fact that HS mice tended to spend less time engaging in HS behavior in response to ESC at the study endpoint, is in support of neurobiological evidence implicating lower cortico-striatal concentrations of serotonin to contribute to excessive behavioral engagement (Clarke et al., 2007). Such a conclusion is further supportive of our other present findings pointing to stereotypy not being temporally associated with an anxiety-like response. Since chronic high-dose SSRI treatment is a known anxiolytic intervention, it stands to reason that if a lower threshold of anxiety sensitivity was promulgating the behavioral expression of HS mice, high dose ESC administered over 4 weeks, would have abrogated the expression of such behavior. Our perspective on the present result is thus one that regards HS expression in deer mice as a repetitive behavioral phenotype that is unique in terms of its neurobiology and, while dissociated from an anxiogenic *cause*, might be associated with a distinct psychobiological fingerprint in HS, compared to NS animals. To this, we afforded more attention in the introduction of this work.

This perspective may also explain why HS, but not NS deer mice exposed to chronic R, showed a lower degree of stereotypical engagement over time (although we observed no post-intervention difference at either sub-acute or chronic level between the control- and R-exposed mice). This result was robust and significant (average highest stereotypical intensity: *d* = 1.0; *p* = 0.024; average percentage time spent engaging in HS behavior: *d* = 1.5; *p* = 0.02), while neither ESC administered on its own or in combination with R, resulted in the same degree of adaptation over time. Mostly in line with the conclusions of Würbel et al. (1998) with respect to wire-gnawing in ICR mice—which argued said behavior to be a behavioral habit—the present results point more to a similar explanation of HS behavior in deer mice than to a motivational cognitive architecture underlying high stereotypy. To explain, rigid behavioral stereotypies that arise in a less complex environment may be related to a lower degree of functional behavioral organization. Thus, and taking into account earlier work that showed HS deer mice to present with perturbations in cognitive performance also (de Brouwer et al., 2021; de Ridder et al., 2022), it is possible that unique interactions between the standard laboratory environment and distinct neurobiological and genetic influences, might contribute to HS animals presenting with a behavioral phenotype that is representative of a lower degree of behavioral organization, as proposed for ICR mice in earlier work (Würbel and Stauffacher, 1996). Still, HS behavior is remarkably similar in its psychobiological resemblance to compulsive-like processes. In fact, instead of refuting the possible involvement of compulsive-like processes in these animals, we propose that studies into HS behavior and the manner of its responding to both behavioral and pharmacological intervention might shed light on the influence of distinct neurocognitive processes, i.e., habitual processing, on the manifestation of certain persistent and repetitive phenotypes, much like these play a distinct role in a unique subset of OCD patients as well (Gillan et al., 2011).

## 5 Conclusion

The present body of work highlights a unique psychobiological signature in HS compared to NS deer mice, in that the manner of anxiety processing in NS and HS deer mice associated with a differential effect of LOR on R-induced behavioral expression. We further conclude that while said anxiety-related differences cannot be temporally linked to the expression of stereotypy, they may be indicative of other parallel cognitive perturbations in HS, compared to NS animals. Last, our results show that HS behavior in deer mice is more representative of a learned behavioral expression which can be unlearned in response to the prevention of its execution. This finding is important and should be explored in terms of its potential to contribute to our understanding of the relationships between compulsivity and other related neurocognitive constructs.

## Data Availability Statement

The raw data supporting the conclusions of this article will be made available by the authors, without undue reservation.

## Ethics Statement

The animal study was reviewed and approved by Animal Care Research Ethics Committee, North-West University.

## Author Contributions

JB, DM, and DW contributed to the conceptualization and design of the investigation. All authors contributed to the interpretation of the results. JB performed all the experimental procedures and data processing. JB and DW performed the statistical analyses and wrote the first draft of the manuscript. All authors contributed to the article and approved the submitted version.
